# Comprehensive analysis identifying aberrant DNA methylation in rectal mucosa from ulcerative colitis patients with neoplasia

**DOI:** 10.18632/oncotarget.26032

**Published:** 2018-09-04

**Authors:** Yuji Toiyama, Yoshinaga Okugawa, Satoru Kondo, Yoshiki Okita, Toshimitsu Araki, Kurando Kusunoki, Motoi Uchino, Hiroki Ikeuchi, Seiichi Hirota, Akira Mitsui, Kenji Takehana, Tsutomu Umezawa, Masato Kusunoki

**Affiliations:** ^1^ Department of Gastrointestinal and Pediatric Surgery, Division of Reparative Medicine, Institute of Life Sciences, Graduate School of Medicine, Mie University, Mie, Japan; ^2^ Department of Inflammatory Bowel Disease, Hyogo College of Medicine, Hyogo, Japan; ^3^ Department of Surgical Pathology, Hyogo College of Medicine, Hyogo, Japan; ^4^ Institute for Innovation, Ajinomoto Co., Inc., Kawasaki, Japan; ^5^ R&D Planning Department, EA Pharma Co., Ltd., Tokyo, Japan

**Keywords:** colitis-associated cancer, methylation, ulcerative colitis

## Abstract

**Background:**

There are no biomarkers to facilitate the identification of patients with ulcerative colitis (UC) who are at high risk for developing colorectal cancer (CRC). In our current study, we used rectal tissues from UC patients to identify aberrant DNA methylations and evaluated whether they could be used to identify UC patients with coexisting colorectal neoplasia.

**Results:**

Using a training set, we identified 484 differentially methylated regions (DMRs) with absolute delta beta-values > 0.1 in rectal mucosa by using the ChAMP algorithm. Next, pathway enrichment analysis was performed using 484 DMRs to select coordinately methylated DMRs, resulting in the selection of 187 aberrant DMRs in rectal tissues from UC-CRC. Then, the Elastic Net classification algorithm was performed to narrow down optimal aberrant DMRs, and we finally selected 11 DMRs as biomarkers for identification of UC-CRC patients. The 11 chosen DMRs could discriminate UC patients with or without CRC in a training set (area under the curve, 0.96) and the validation set (area under the curve, 0.81).

**Conclusions:**

In conclusion, we identified 11 DMRs that could identify UC patients with CRC complications. Prospective studies should further confirm the validity of these biomarkers.

**Methods:**

We performed genome-wide DNA methylation profiles in rectal mucosal tissues (*n* = 48) from 24 UC-CRC and 24 UC patients in a training set. Next, we performed comprehensive DNA methylation analysis using rectal mucosal tissues (*n* = 16) from 8 UC-CRC and 8 UC patients for validation.

## INTRODUCTION

Patients with long-standing ulcerative colitis (UC) are at a higher risk than the general population for developing colorectal cancer (CRC). The prevalence of colitis-associated cancer (CAC) in patients with UC is 8% 20 years after the initial UC diagnosis and increases to 18% at 30 years [1]. CAC is a major cause of mortality in patients with UC [2, 3], so that diagnosis at an early or precancerous stage is crucial.

Surveillance colonoscopy with multiple random biopsies has been widely recommended for patients with long-standing and extensive UC [4]. However, the low yield and lack of clinical consequences from random biopsies in this high-risk population raise questions about the necessity and cost-effectiveness of such UC surveillance [5]. A recent randomized controlled trial to compare rates of neoplasia detected by targeted vs random biopsies in patients with UC from Japan revealed that these methods detected similar proportions of neoplasias. However, a targeted biopsy approach appears to be a more cost-effective method [6].

More accurate diagnostic modalities, such as chromoendoscopy and magnifying endoscopy, to identify potential sites of neoplasia in a non-neoplastic inflamed epithelium, together with analysis of p53 alterations, to distinguish neoplastic lesions from regenerative epithelium, have been evaluated [7, 8]. However, the labor-intensive nature and expense of these adjunctive modalities preclude their use in the surveillance of all UC patients with long-standing and extensive colitis. We suggest that within this subgroup of patients, the ability to distinguish those who are at low vs. high risk of colorectal neoplasia would allow physicians to identify those patients most likely to benefit from these more extensive screening methods.

Recently, we reported that methylation of specific miRNAs (*MIR1, MIR9, MIR124, MIR137* and *MIR34B/C*) occurred in an age- and cancer-dependent manner in UC patients, and that methylation of these 5 miRNAs in non-neoplastic rectal mucosa successfully discriminated patients with UC-CRC from those without in 2 independent patient cohorts [9]. However, the study had limitations because it focused on methylation of aging- and cancer-associated miRNAs. Therefore, further studies including a broader, unbiased genome-wide analysis may potentially identify additional methylation loci to assess for the risk for UC-CRC.

In the current study, we examined the possibility of using genome-wide DNA methylation array analysis of rectal tissues of UC patients to identify aberrant DNA methylation and thereby identify UC patients who had coexisting colorectal neoplasia.

## RESULTS

### Identification of differentially methylated regions in rectal mucosal tissues of UC-associated colorectal cancer

**Figure 1:**
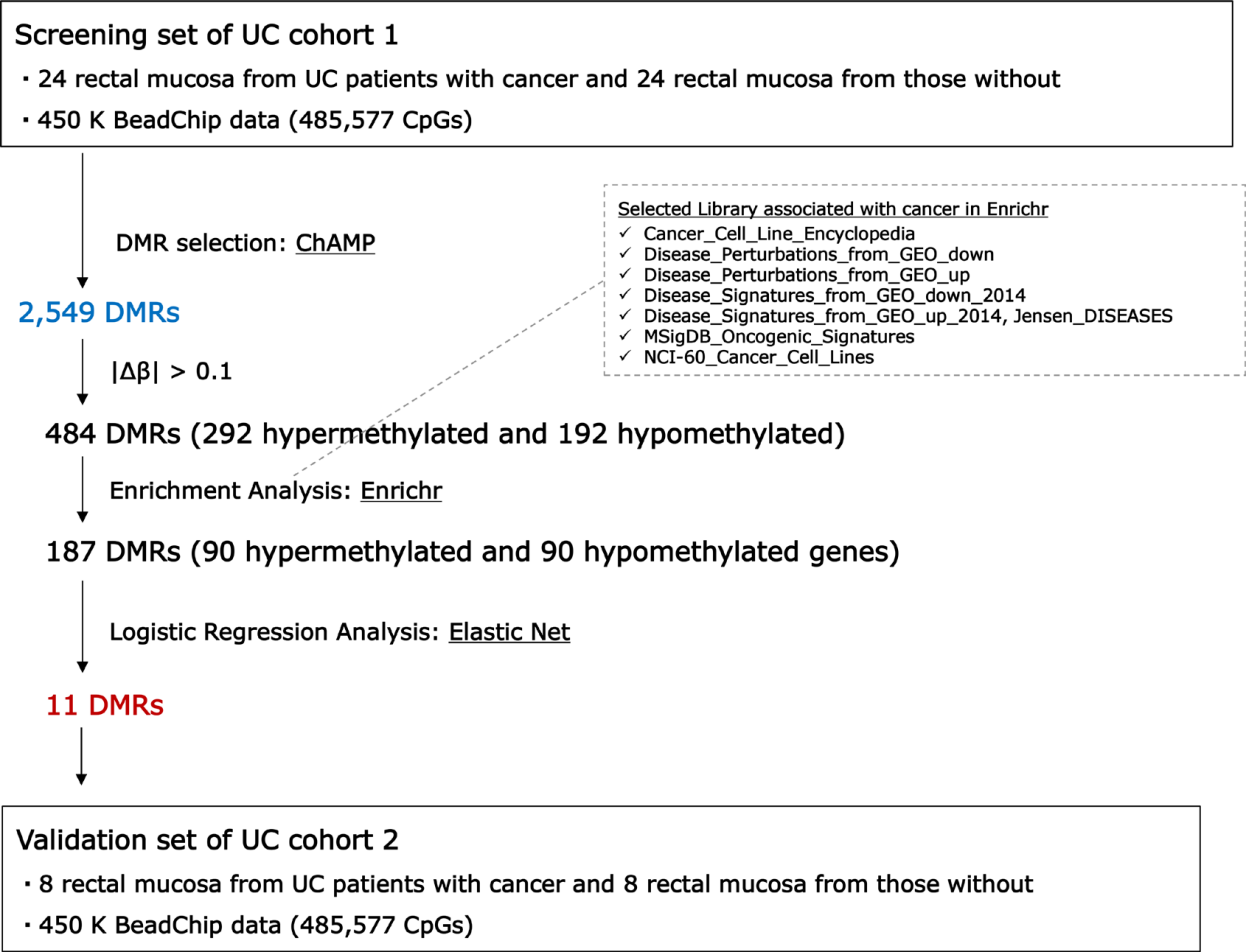
Workflow of differentially methylated regions (DMRs) selection

**Figure 2:**
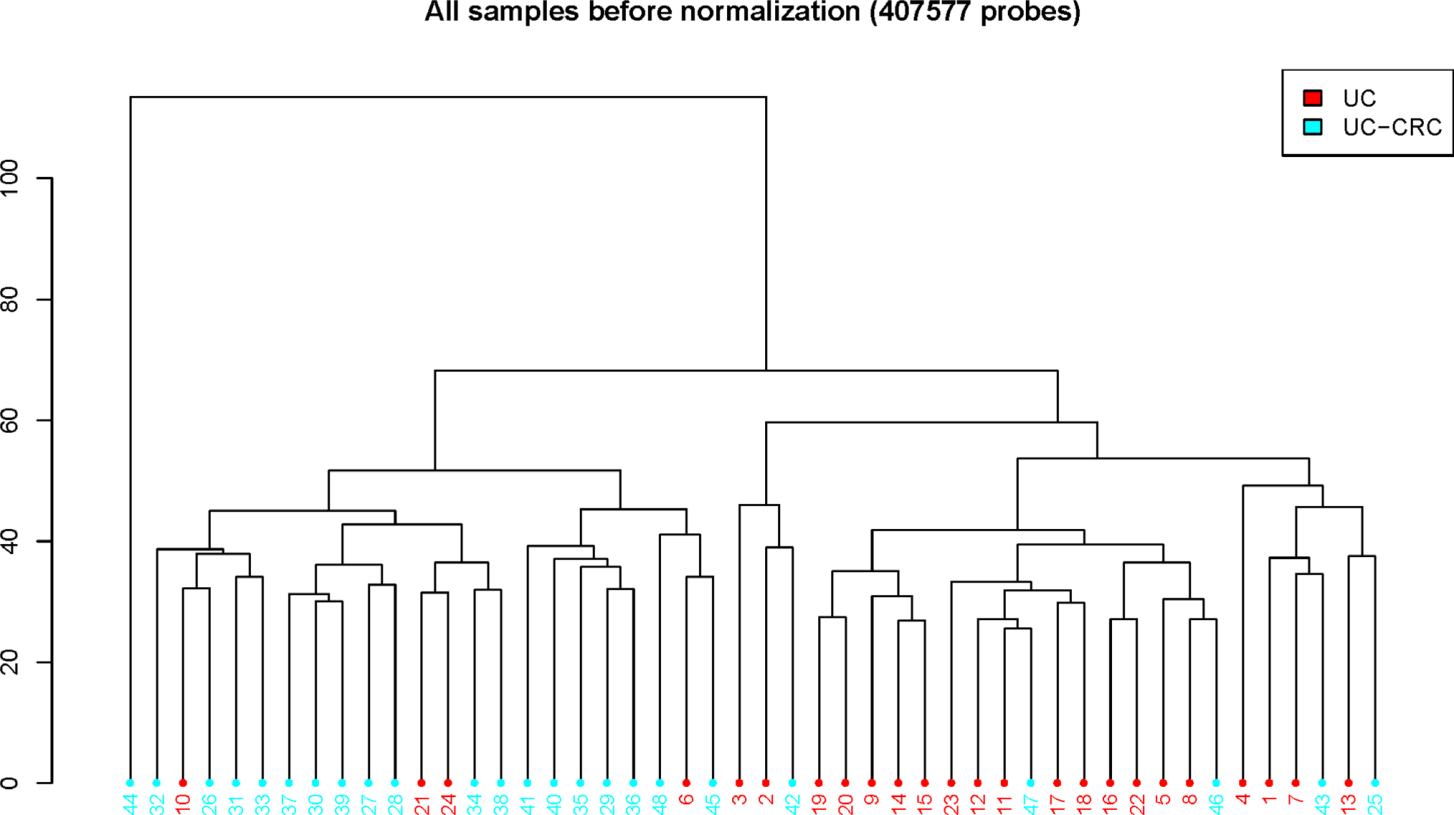
Hierarchical clustering of UC-CRC and UC data ChAMP package generated the plot using 407,577 probes.

We used the Infinium HumanMethylation450 BeadChip to compare genome-wide DNA methylation profiles in rectal mucosal tissues of UC-CRC and UC patients. On average, 484,961 CpGs were detected. Figure 1 shows the overall workflow for selection of differentially methylated regions (DMR). By using the ChAMP package, we identified 15,478 differentially methylated probes that lay in 2,549 DMRs [10], 9,759 (63.1%) being hypermethylated and 5,719 (36.9%) being hypomethylated. Of the 15,478 probes, 5,027 (32.5%), 1,960 (12.7%), 1,374 (8.9%), 1,350 (8.7%), 496 (3.2%) and 460 (3.0%) were located in the gene body, TSS1500 (within 1,500 base pairs upstream or downstream of the transcriptional start site), TSS200 (within 200 base pairs upstream or downstream of the transcriptional start site), 5′-UTR, 3′-UTR and the first exon, respectively. The remainder (4,811, 31.1%) were in intergenic regions (IGR). Of 484 DMRs with absolute delta beta-values > 0.1, 292 DMRs were hypermethylated and 192 DMRs were hypomethylated. By inspection of the result of hierarchical clustering analysis, one outlier sample (UC-CRC #44) was excluded from further analysis (Figure 2).

### Pathway enrichment analysis

The 484 DMRs were subjected to a gene set enrichment analysis in order to select coordinately methylated DMRs. We assumed that coordinately methylated genes would have biologically significant roles and provide potentially robust diagnostic power. We searched for the overrepresented biological pathways associated with the differentially methylated genes using the Enrichr analysis tool [11]. The 464 genes associated with 484 DMRs were used as the input, 268 being hypermethylated and 196 being hypomethylated. Results for the enrichment analysis are shown in Tables 1 and 2. Enrichr’s combined score, a combination of the *P* value and z-score, was used to prioritize enriched pathways.

**Table 1:** Enrichr terms significantly enriched with differentially methylated genes

Term	Overlap	*P* value	Adjusted *P* value	*Z* score	Combined score	Genes	Library
Cleft_palate	8/98	5.2E-05	9.8E-03	–3.68	36.3	*TFAP2A, COL2A1, SATB2, WNT5A, PAX9, HOXA2, VAX1, MEIS2*	Jensen_DISEASES
Renal_agenesis	5/27	2.6E-05	9.8E-03	–2.81	29.7	*SALL1, GDNF, SATB2, HOXA2, HS2ST1*	Jensen_DISEASES
SIMA_AUTONOMIC_GANGLIA	19/444	9.5E-06	7.7E-03	–1.88	21.8	*TFAP2D, KCNJ8, SLC7A14, SOX11, ESRRG, TMEM196, REC8, ST18, TTLL6, DPP6, FGF14, DAB1, TLX3, HAND2, NPY, RADIL, HOXD3, CYP2E1, HOXD8*	Cancer_Cell_Line_ Encyclopedia
Spinal Muscular Atrophy C0026847 mouse GSE10599 sample 235	17/368	1.1E-05	7.5E-03	–1.70	19.5	*C11ORF87, PTPRN2, SLC32A1, SLC7A14, GRIK2, SEZ6L2, GABRG3, SLC6A5, CLDN11, BCAN, DPP6, PRKAR1B, CHL1, SLITRK1, SPOCK1, AMPH, OPCML*	Disease_Perturbations_ from_GEO_up
NCIH2141_LUNG	15/327	3.8E-05	1.3E-02	–1.74	17.7	*TMEM132D, KHDRBS2, SOX11, ESPN, HOXD10, REC8, ST18, MTMR7, NELL1, PEX5L, DPP6, ABLIM2, FGF14, COL2A1, SP8*	Cancer_Cell_Line_ Encyclopedia
NCIH1092_LUNG	16/396	9.5E-05	1.3E-02	–1.90	17.6	*TMEM132D, SLC32A1, SLC7A14, SOX11, ESRRG, HOXD10, ST18, NELL1, PEX5L, DPP6, ABLIM2, FGF14, AMPH, SP8, SDCCAG8, HOXB5*	Cancer_Cell_Line_ Encyclopedia
NCIH1184_LUNG	14/300	5.7E-05	1.3E-02	–1.79	17.4	*TMEM132D, KHDRBS2, SLC32A1, TMEM196, TBX3, ST18, DPP6, FGF14, COL2A1, RIMS3, RFX4, NPY, ZNF568, CDO1*	Cancer_Cell_Line_ Encyclopedia
NCIH1930_LUNG	14/310	8.1E-05	1.3E-02	–1.75	16.5	*SLC32A1, NR2E1, ST18, NELL1, PLCXD3, DPP6, FGF14, CHL1, RFX4, CHL1-AS2, RADIL, HOXD3, PDZRN4, HOXD8*	Cancer_Cell_Line_ Encyclopedia
NCIH211_LUNG	12/235	8.3E-05	1.3E-02	–1.75	16.4	*TMEM132D, UVRAG, SIX2, RFX4, NPY, ZFP42, TNFRSF25, ZNF568, OLIG2, ESPN, TBX3, MTMR7*	Cancer_Cell_Line_ Encyclopedia
DMS79_LUNG	15/367	1.4E-04	1.6E-02	–1.81	16.1	*TMEM132D, SLC32A1, SLC7A14, ESPN, GABRG3, TMEM196, TBX3, MTMR7, CCNA1, ABLIM2, FGF14, RIMS3, PAX9, CAMTA1, PDZRN4*	Cancer_Cell_Line_ Encyclopedia
NCIH209_LUNG	12/265	2.5E-04	2.6E-02	–1.75	14.5	*NELL1, PEX5L, DPP6, ABLIM2, FGF14, RIMS3, PSMB2, ESPN, HOXD10, HOXD8, ST18, MTMR7*	Cancer_Cell_Line_ Encyclopedia
KPNYN_AUTONOMIC_ GANGLIA	13/326	4.8E-04	3.9E-02	–1.82	13.9	*SLC7A14, SOX11, ESRRG, NELL1, PLCXD3, DPP6, FGF14, SLITRK1, HAND2, ADAMTS17, RADIL, CDO1, GRIA4*	Cancer_Cell_Line_ Encyclopedia
MOGGCCM_CENTRAL_ NERVOUS_SYSTEM	7/112	7.9E-04	4.8E-02	–1.94	13.8	*SFRP2, DAB1, CA3, PCDHGC3, HOXA2, MDFI, OPCML*	Cancer_Cell_Line_ Encyclopedia
KNS42_CENTRAL_ NERVOUS_SYSTEM	10/193	2.9E-04	2.6E-02	–1.69	13.8	*ACSS3, WBSCR17, SFRP2, SALL1, CHL1, RFX4, ZIC1, SP8, NR2E1, CDO1*	Cancer_Cell_Line_E ncyclopedia
WM1799_SKIN	8/143	7.0E-04	4.7E-02	–1.78	13.0	*NELL1, TFAP2E, GDNF, HPSE2, CHL1, ABCB5, CHL1-AS2, ALX1*	Cancer_Cell_Line_ Encyclopedia
TE441T_SOFT_TISSUE	11/248	5.4E-04	4.0E-02	–1.72	12.9	*DPP6, DAB1, SALL1, COL2A1, ZIC1, WNK4, ALX1, OLIG2, CDO1, ACSF2, GLI3*	Cancer_Cell_Line_ Encyclopedia
CHP126_AUTONOMIC_ GANGLIA	15/435	8.3E-04	4.8E-02	–1.76	12.5	*KCNJ8, SLC7A14, SOX11, TMEM196, NELL1, WBSCR17, DPP6, RIMS3, TLX3, SLITRK1, HAND2, ADAMTS17, HOXD3, HOXD8, GRIA4*	Cancer_Cell_Line_ Encyclopedia
IMR32_AUTONOMIC_ GANGLIA	12/307	9.4E-04	4.8E-02	–1.77	12.3	*WBSCR17, DPP6, RIMS3, KCNJ8, SLITRK1, SIX2, HAND2, SLC7A14, SOX11, ESRRG, RADIL, HOXD3*	Cancer_Cell_Line_ Encyclopedia
SKNFI_AUTONOMIC_ GANGLIA	13/349	9.1E-04	4.8E-02	–1.75	12.3	*PCDH15, SLC7A14, SOX11, GRIK2, TBX3, MTMR7, DPP6, RIMS3, GDNF, CHL1, HAND2, NPY, CHL1-AS2*	Cancer_Cell_Line_ cyclopedia

268 hypermethylated genes as the input. Terms are ordered according to the combined scores.

**Table 2:** Enrichr terms significantly enriched with differentially methylated genes

Term	Overlap	*P* value	Adjusted *P* value	*Z* score	Combined score	Genes	Library
HPAFII_PANCREAS	11/129	5.9E-08	2.7E-05	–1.87	31.1	*LGALS4, SULT2B1, ARL14, HNF4A, CDHR2, TRIM15, OVOL1, MUC20, MUC4, NCKAP5, SGK2*	Cancer_Cell_Line_ Encyclopedia
OE19_OESOPHAGUS	15/272	8.1E-08	2.7E-05	–1.73	28.2	*GRB7, PLA2G4F, CANT1, IDUA, C9ORF152, LTBP4, LGALS4, SULT2B1, BAIAP2L2, IYD, MCF2L, HNF4A, SPIRE2, TFF1, SGK2*	Cancer_Cell_Line_ Encyclopedia
colon cancer DOID-219 human GSE34299 sample 502	15/304	3.4E-07	2.6E-04	–1.79	26.7	*ARL14, LAMB3, MTMR11, TMPRSS4, PDGFA, C15ORF52, C4BPB, PPP2R3A, SERPINB5, SULT2B1, ARL4A, IFI27, S100A16, MALL, SNX9*	Disease_Perturbations_ from_GEO_up
2313287_STOMACH	11/159	4.9E-07	1.1E-04	–1.81	26.3	*BAIAP2L2, PARD6B, MTMR11, C9ORF152, IYD, PPP1R16A, SPIRE2, NOSTRIN, TRIM15, TFF1, CARD14*	Cancer_Cell_Line_ Encyclopedia
SNU16_STOMACH	10/153	2.8E-06	4.6E-04	–1.84	23.5	*LGALS4, MUC2, CDX1, C9ORF152, IYD, HNF4A, NOSTRIN, TRIM15, SGK2, GALNT8*	Cancer_Cell_Line_ Encyclopedia
HCC2998	15/335	1.2E-06	1.0E-04	–1.70	23.2	*ST14, CDX1, ANKRD11, ATP11A, ADAP1, OVOL1, LGALS4, BAIAP2L2, IFI27, INPP5J, S100P, TRIM15, PKP3, PRSS8, GALNT8*	NCI-60_Cancer_Cell_ Lines
Barrett’s esophagus DOID-9206 human GSE34619 sample 596	13/311	1.3E-05	3.3E-03	–1.65	18.6	*ARL14, C9ORF152, C12ORF75, LGALS4, SELENBP1, FAM134B, IFI27, CDHR2, NOSTRIN, TFF1, MIR192, SGK2, MISP*	Disease_Perturbations_ from_GEO_up
Barrett’s esophagus DOID-9206 human GSE34619 sample 453	13/309	1.2E-05	3.3E-03	–1.63	18.4	*ARL14, C9ORF152, C12ORF75, LGALS4, SELENBP1, FAM134B, MUC2, CDHR2, NOSTRIN, TFF1, MIR192, SGK2, MISP*	Disease_Perturbations_ from_GEO_up
NCIH508_LARGE_INTESTINE	11/258	5.0E-05	5.6E-03	–1.86	18.4	*LGALS4, PLA2G4F, MUC2, ADPRHL1, CDX1, HNF4A, INPP5J, SPIRE2, TRIM15, PLCH2, RNF186*	Cancer_Cell_Line_ Encyclopedia
SNU520_STOMACH	13/333	2.6E-05	3.5E-03	–1.74	18.3	*CASZ1, C9ORF152, PPP1R16A, LGALS4, BAIAP2L2, MUC2, IYD, CDHR2, SPIRE2, NOSTRIN, TRIM15, SGK2, FRK*	Cancer_Cell_Line_ Encyclopedia
pancreatic ductal adenocarcinoma DOID-3498 human GSE15471 sample 604	15/487	9.6E-05	1.1E-02	–1.87	17.3	*ARL14, LAMB3, MTMR11, TMPRSS4, C15ORF52, SERPINB5, LGALS4, C1ORF106, COL3A1, IFI27, ELF3, FXYD3, APOD, TFF1, S100P*	Disease_Perturbations_ from_GEO_up
NCIH854_LUNG	12/332	1.1E-04	1.1E-02	–1.81	16.5	*LGALS4, C1QTNF1, MUC2, C9ORF152, MCF2L, HNF4A, B3GALT2, LRP5, LTBP4, TRIM15, TFF1, LINC00112*	Cancer_Cell_Line_ Encyclopedia
OVCAR5	9/180	7.3E-05	3.2E-03	–1.70	16.2	*ST14, ARL14, DOCK9, MALL, TNFAIP2, AGAP1, OVOL1, MUC4, EXPH5*	NCI-60_Cancer_Cell_ Lines
Barrett’s esophagus DOID-9206 human GSE1420 sample 643	12/314	6.6E-05	1.1E-02	–1.68	16.2	*LGALS4, LGALS3BP, C1ORF106, COL3A1, MUC2, IFI27, LAMB3, FXYD3, TFF1, S100P, MISP, SERPINB5*	Disease_Perturbations_ from_GEO_up
Adenocarcinoma of esophagus C0279628 human GSE1420 sample 164	12/318	7.5E-05	1.1E-02	–1.69	16.0	*LGALS4, IL32, LGALS3BP, COL3A1, MUC2, IFI27, LAMB3, ELF3, TFF1, S100P, MISP, SOD3*	Disease_Perturbations_ from_GEO_up
TCCPAN2_PANCREAS	7/120	1.8E-04	1.5E-02	–1.85	16.0	*LGALS4, ADPRHL1, HNF4A, CDHR2, C4BPB, SOD3, SGK2*	Cancer_Cell_Line_ Encyclopedia
cystic fibrosis DOID-1485 human GSE15568 sample 833	11/278	9.8E-05	1.1E-02	–1.72	15.9	*LGALS4, LGALS3BP, MUC2, IFI27, TPM4, MALL, TFF1, S100P, PRSS8, MISP, SERPINB5*	Disease_Perturbations_ from_GEO_up
NCIH1435_LUNG	6/108	6.9E-04	4.1E-02	–1.87	13.6	*SULT2B1, HDAC4, DOCK9, IYD, TRIM15, CARD14*	Cancer_Cell_Line_ Encyclopedia
C3A_LIVER	12/387	4.6E-04	3.3E-02	–1.74	13.4	*SLC26A1, F10, MOGAT2, HNF4A, CDHR2, GAS2, LINC00479, TRIM15, C4BPB, AGXT, SLC30A10, SERPINA6*	Cancer_Cell_Line_ Encyclopedia
esophagus adenocarcinoma DOID-4914 human GSE1420 sample 644	11/322	3.5E-04	3.4E-02	–1.66	13.2	*LGALS4, IL32, LGALS3BP, COL3A1, MUC2, IFI27, LAMB3, TFF1, S100P, MISP, SOD3*	Disease_Perturbations_ from_GEO_up
JHOM2B_OVARY	8/189	5.7E-04	3.7E-02	–1.76	13.2	*LGALS4, SULT2B1, CDX1, IYD, TRIM15, RNF186, MUC20, SGK2*	Cancer_Cell_Line_ Encyclopedia
allergic asthma DOID-9415 human GSE41649 sample 716	11/329	4.2E-04	3.6E-02	–1.69	13.2	*IL32, COL3A1, MUC2, C1QTNF1, IFI27, NOS2, TMPRSS4, ERI3, S100P, MUC4, CAPN15*	Disease_Perturbations_ from_GEO_up
COLO205	12/421	9.5E-04	2.8E-02	–1.57	10.9	*LGALS4, HDAC4, BAIAP2L2, CANT1, ELF3, TRIM2, CDHR2, TMPRSS4, S100P, ATP11A, SGK2, SERPINB5*	NCI-60_Cancer_Cell_ Lines
KM12	7/179	2.0E-03	4.3E-02	–1.60	10.0	*CDX1, HNF4A, S100P, TRIM15, TFF1, PLCH2, SERPINB5*	NCI-60_Cancer_Cell_ Lines

196 hypomethylated genes as the input. Terms are ordered according to the combined scores.

The hypermethylated genes were associated with 19 enrichment terms, including cancer cell lines of lung, ganglia and central nervous system and skin (Table 1). The hypomethylated genes were associated with 24 enrichment terms, including cancer cell lines as well as terms in the Disease_Permutaions_from_GEO_up library (Table 2). Colon cancer and adenocarcinomas are included in the library and all of the genes in these terms are categorized as upregulated. We extracted genes that appeared at least once in the enrichment terms. Of these, 90 genes were hypermethylated and 90 were hypomethylated. DMRs (187) were associated with these genes and used as input for the next step.

### Logistic regression analysis

**Figure 3:**
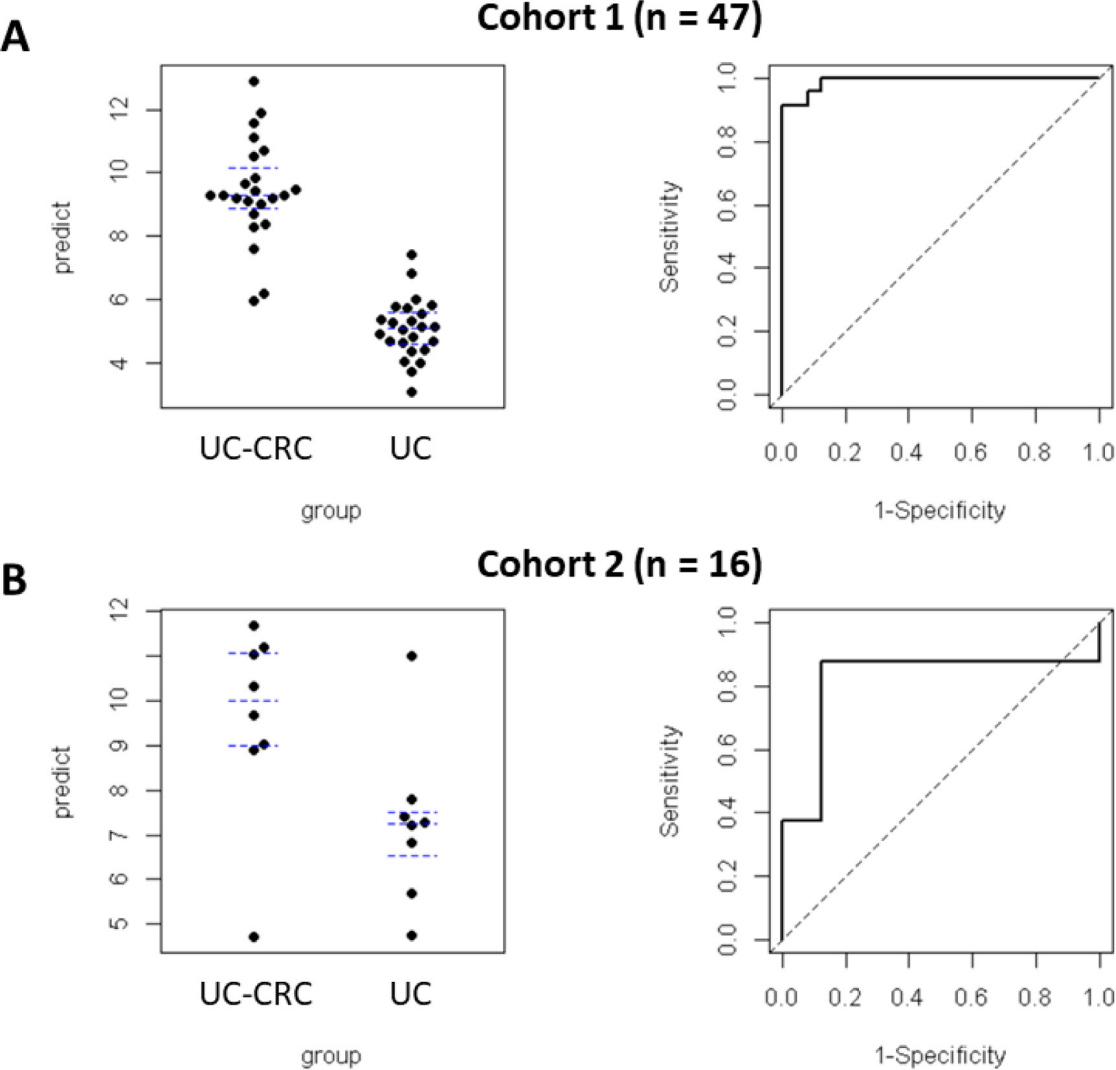
Predictive values and ROC curves quantifying the performance of Elastic Net regularized logistic regression model (glmnet) in predicting UC-CRC Predictive values were plotted (left panels: (**A**), Cohort 1; (**B**), Cohort 2). ROC curves were plotted for both cohorts. The AUC for the training set (Cohort 1: A, right panel) was 0.96 (95% CI: 0–90, 1.00). The AUC for the validation set (Cohort 2: B, right panel) was 0.81 (95% CI: 0.55, 1.00). The regression coefficients obtained from the training set were also applied to the validation set.

We applied the Elastic Net classification algorithm to choose DMRs [12]. Of the 187 DMRs, 11 were retained in all 100 runs in the training set (*n* = 47, Cohort 1). Ten DMRs (*SIX2, SATB2/SATB2-AS1, HAND2, GDNF, PLCXD3, HPSE2, TBX3, PAX9, MEIS2* and *SALL1*) were hypermethylated and 1 DMR (*LGALS3BP*) was hypomethylated (Table 3). In the ROC analysis for the training set (*n* = 47, Cohort 1), the AUC was 0.96 (95% CI: 0.90, 1.00) (Figure 3). For the validation set (*n* = 16, Cohort 2), the AUC was 0.81 (95% CI: 0.55, 1.00). Supplementary Table 1 showed the methylation levels of 11DMRs in non-neoplastic tissues and neoplastic tissues UC-CRC patients in validation set, and the aberrant methylation of 11 DMRs was more remarkable in neoplastic tissues than in non-neoplastic tissues of UC-CRC.

**Table 3:** 11 DMRs selected using Elastic Net classification algorithm

Gene	dmrChrom	dmrStart	dmrEnd	dmrSize	betaUC	betaUC-CRC	deltaBeta
*SIX2*	chr2	45,233,485	45,233,784	300	0.45	0.66	0.21
*SATB2;SATB2-AS1*	chr2	200,334,655	200,335,051	397	0.12	0.36	0.24
*HAND2*	chr4	174,444,434	174,447,969	3,536	0.53	0.65	0.12
*GDNF*	chr5	37,835,048	37,835,287	240	0.32	0.52	0.20
*PLCXD3*	chr5	41,509,803	41,509,960	158	0.45	0.58	0.13
*HPSE2*	chr10	100,993,404	100,994,628	1,225	0.17	0.31	0.13
*TBX3*	chr12	115,130,993	115,135,866	4,874	0.34	0.47	0.13
*PAX9*	chr14	37,124,148	37,124,479	332	0.33	0.46	0.13
*MEIS2*	chr15	37,387,445	37,387,655	211	0.36	0.52	0.16
*SALL1*	chr16	51,168,402	51,168,636	235	0.24	0.38	0.14
*LGALS3BP*	chr17	76,976,153	76,976,472	320	0.67	0.55	–0.13

## DISCUSSION

It has been proposed that in UC patients, chronic inflammation increases epithelial cell turnover in the colonic mucosa, resulting in the acquisition of genetic and epigenetic alternations in non-neoplastic mucosa during cancer development [13–16]. In fact, previous studies have demonstrated that patients with UC-CRC have widespread genetic alterations in non-neoplastic colonic mucosa [17–20]. These results suggested that the detection of molecular changes in non-neoplastic mucosa could provide biomarkers for predicting the risk associated with CRC in UC patients. However, they have not been confirmed by subsequent studies and are routinely used in the clinical setting.

On the other hand, inflammation in UC patients characteristically begins in the rectal mucosa and spreads progressively and contiguously to the proximal colon [21]. We suggest that non-neoplastic rectal mucosa might be the optimal site to investigate aberrant molecular changes during carcinogenesis. Few studies of rectal mucosa have been conducted to search for predictive markers capable of identifying high risk UC patients with developing CRC. Watanabe *et al.* used microarray analysis of rectal mucosa from UC patients to identify a gene expression signature that was predictive of the development of UC-associated neoplasm [22]. We found 5 miRNAs to be hypermethylated in rectal mucosa from UC patients with dysplasia or CRC compared with patients without neoplasms, and they might be used to identify patients with UC at greatest risk for developing UC-CRC [9]. However, comprehensive high-throughput analysis of methylation status in rectal mucosa has not been reported.

In the current study, we undertook the first comparison of differential DNA methylation profiles in rectal mucosa between UC patients with CRC and those without using the Illumina HumanMethylation450 BeadChip. Then, we narrowed down specific DMRs by statistical analyses using several algorithms. Finally, we searched for a set of DMRs that were significantly different in rectal mucosa from UC-CRC in cohort 1. The 11 DMRs that we identified in rectal mucosa were robust in discriminating UC patients with CRC from those without, with AUC values of 0.96 (95% CI: 0.90, 1.00). Thereafter, 11 DMRs were successfully validated in an independent set of rectal samples from UC patients. In addition, the sensitivity and specificity were high in both the training set and validation cohort, as expected. Thus, our results suggest that analysis of the status of 11 DMRs in a single rectal biopsy could help identify UC patients that are at greatest risk of developing neoplasia, which would be a substantially more practical strategy in contrast to current surveillance protocols.

Pathway analysis identified DMR-associated enrichment terms. The hypomethylated genes were associated with 24 enrichment terms, including cancer cell lines as well as terms in the Disease_Permutaions_from_GEO_up library. Colon cancer [23] and adenocarcinomas [24, 25] are included in the library and all of the genes in these terms are categorized as upregulated. Hypomethylation of these DMRs would be associated with upregulation of corresponding genes. However, we did not perform gene expression analysis due to the limitation of FFPE-derived RNA samples. Therefore, further study should be conducted to investigate the association between DNA methylation status on selected DMRs and expression of their genes.

Our Elastic Net classification algorithm selected 11 DMRs. The aberrant methylation of 11 DMRs was more remarkable in neoplastic tissues than in non-neoplastic tissues, which suggest that these genes are associated with carcinogenesis in colonic mucosa. Of these, heart and neural crest derivatives expressed 2 genes (*HAND2* and *SALL1*) reported to be hypermethylated and associated with tumorigenesis in tumor tissues. HAND2 is a basic helix-loop-helix transcription factor that plays a very important role in the development and differentiation of the heart and nervous system [26]. Recent studies revealed that *HAND2* was significantly hypermethylated and downregulated in colon and rectal cancer [27, 28]. In addition, continuous proliferation of the endometrium was observed in mice with knockdown of *HAND2* [29]. Here, our bioinformatic results showed that *HAND2*’s methylation status of rectal mucosa was dysregulated in UC patients with CRC, which was first reported. Collectively, it is suggested that *HAND2* has characteristics of tumor suppressor genes in several types of tumors.

SALL1 is a multi-zinc finger transcription factor that regulates organogenesis and stem cell development [30]. In breast cancer, SALL1 acts as a tumor suppressor that recruits the NuRD complex and thereby induces cell senescence [30]. In addition, inhibition of SALL1 correlates with reduced levels of CDH1, an important contributor to the epithelial-to-mesenchymal transition [31].

GDNF was also selected as one of the 11DMRs. The GDNF family of ligands and their receptors activate the Ret signaling pathway and regulate cell survival and proliferation [32]. In addition, Ret expression compromises neuronal cell survival in the colon [33]. Therefore, it has been postulated that GDNF is a novel member in the set of protective mucosal factors [34]. In this context, dysregulation of GDNF could lead to down-regulation of Ret expression and may finally result in failure of colonic mucosal protection. Saito *et al.* [35] demonstrated that increased methylation of *CDH1* and *GDNF* is correlated with severe inflammation in the colonic mucosa of UC, which indicates a potential epigenetic mechanism underlying mucosal inflammation and occurrence of dysplasia/cancer with chronic inflammation in UC patients.

In conclusion, in screening a patient cohort, we successfully selected 11 DMRs that could identify UC patients that would progress to developing CRC. This was achieved by whole-genome methylation analysis followed by Pathway enrichment analysis and Elastic Net regularized regression modeling. Furthermore, whole-genome methylation data from a validation cohort confirmed the 11 DMRs to be promising biomarkers in UC with CRC. Although our findings were successfully validated with an external independent cohort, the number of patients with UC was still limited, and longitudinally collected rectal specimens from UC patients were not available in this study. Therefore, larger prospective studies will be needed to confirm the validity of these predictors. However, we believe the analysis of these 11DMRs from a single rectal biopsy specimen might have robust predictive potential in permitting the identification of UC patients that are at high risk for neoplasia elsewhere in the colorectum.

## MATERIALS AND METHODS

### Patients and samples

This study analyzed a total of 64 non-neoplastic rectal epithelial specimens that were obtained from 64 patients diagnosed with UC from 2 different patient cohorts enrolled at Hyogo College of Medicine and Mie University in Japan. In the training set, 48 non-neoplastic rectal epithelial specimens were collected from 48 UC patients with (*n* = 24) or without dysplasia or cancer (*n* = 24). All formalin-fixed paraffin-embedded (FFPE) samples were retrieved from colectomy specimens that were collected at the Hyogo College of Medicine between 2005 and 2011 (Supplementary Table 2). There was no significant difference between the UC patients with cancer and without in training cohort. In the validation cohort, 16 non-neoplastic rectal epithelial specimens were collected from 16 UC patients with (*n* = 8) or without dysplasia or cancer (*n* = 8) (Supplementary Table 3). All tissues were also FFPE and were retrieved from colectomy specimens resected at the Mie University Hospital between 2005 and 2015. Although significant difference was not recognized in validation cohort, it had a trend to increase disease duration, and to decreases disease severity in UC patients with cancer. Specimen collection and studies were approved by the Institutional Review Board of all participating institutions. All participants provided written informed consent and willingness to donate their tissue samples for research.

The diagnosis of UC was based on medical history, endoscopic findings, histologic examination, laboratory tests, and clinical disease presentation. Patients who presented with their first attacks, or infectious colitis caused by *C. difficile* or cytomegalovirus were excluded from this study.

### DNA extraction from FFPE samples

FFPE tissue blocks were serially sectioned at a thickness of 10 μm. Based on histologic findings, mucosal tissues from each region were micro-dissected and genomic DNA was extracted using the QIAamp DNA FFPE tissue kit (Qiagen) according to the manufacturer’s instructions.

### Methylation analysis

Whole-genome DNA methylation profiles were quantified using the Infinium HumanMethylation450 BeadChip Array (Illumina), which measures 485,577 CpG sites at Riken Genesis Co., Ltd., Japan. Prior to the BeadChip Array analysis, quality control of FFPE DNA was performed using the Illumina FFPE QC Kit and Fast SYBR Green Master Mix. Amplified fluorescence was measured using a Step One Plus Real-Time PCR System. The Ct value of each sample was determined and the differences between sample and positive control (delta Ct) were measured. Samples with delta Ct below 5 were passed and bisulfite treated using the EZ DNA Methylation Kit (D5004; Zymo Research, Inc., Irvine, CA, USA). To repair damaged DNA, the Infinium HD FFPE restore Kit was used. The repaired DNA was isothermally amplified overnight at 37° C, followed by an enzymatic fragmentation step. The fragmented DNA was precipitated, resuspended and loaded on the 12-sample BeadChip that was then incubated overnight at 48° C, allowing the fragmented DNA to hybridize to locus-specific 50-mers. Non-specifically hybridized DNA was washed away, followed by a single-base extension reaction using DNP- and biotin-labeled ddNTPs (with the use of a Tecan EVO robot). Subsequently, hybridized DNA was removed from the labeled oligonucleotides and chips were dried under vacuum and imaged using an Illumina iScan device. Data were extracted using GenomeStudio (Illumina, Methylation Module v1.9), which was also used to subtract the background and to normalize staining intensities using internal controls present on the chip. A beta-value was calculated to estimate the methylation level of each CpG locus using the ratio of intensities between methylated and unmethylated alleles (0 = unmethylated, 1 = fully methylated).

### Statistical analysis

#### Identification of differentially methylated regions

Differentially methylated regions (DMRs) were identified using the ChAMP methylation analysis package in R. Briefly, intensity data from IDAT files were loaded and normalized using default settings (i.e., beta-mixture quantile normalization; BMIQ), after which methylation variable positions (MVPs) were identified using R package limma to compare 2 groups. DMRs were identified using an algorithm “probe lasso” implemented in the ChAMP package. DMRs were defined as regions containing 3 or more adjacent probes within a region showing unidirectional changes in methylation that attained nominal significance (unadjusted *p* < 0.05) in the MVP analysis. The lasso region was set to 2 kb and was scaled according to the local genomic/epigenomic landscape in order to account for uneven probe spacing across the genome.

#### Pathway enrichment analysis

Pathway analysis of differentially methylated genes was performed using enrichR, which provides an R interface to all Enrichr databases [11], a web-based tool for analyzing gene sets and returns any enrichment of common annotated biological functions. Enrichr currently contains annotated gene sets from 128 gene set libraries organized in eight categories. We used Cancer_Cell_Line_Encyclopedia, Disease_Perturbations_from_GEO_up, Disease_Perturbations_from_GEO_down, Disease_Signatures_from_GEO_up_2014, Disease_Signatures_from_GEO_down_2014, Jensen_DISEASES, MSigDB_Oncogenic_Signatures and NCI-60_Cancer_Cell_Lones gene set libraries to identify coordinately methylated genes. We considered “terms” in these libraries as enriched if their adjusted *p* value was lower than 0.05 and select genes included in the extracted terms.

#### Logistic regression analysis

Average beta-values of CpG sites in each DMR were calculated and used to build an Elastic Net regularized regression model using the glmnet package in R. Elastic Net is a generalized linear model that operates as a mix of ridge regression and LASSO, which was specifically designed to overcome issues of large variable numbers and small sample size [36]. To account for the randomness of the procedure, we performed it 100 times [37]. After running the 100 iterations, we selected the subset of DMRs that appeared in all 100 to choose a robust subset of DMRs that might be more applicable to other studies. Receiver operating characteristic (ROC) analysis was performed and we calculated the area under the curve (AUC) using pROC package in R.

## SUPPLEMENTARY MATERIALS TABLES



## Abbreviations

AUC: area under the ROC curve
CRC: colorectal cancer
FFPE: formalin-fixed paraffin-embedded
ROC: received operating characteristic
UC: ulcerative colitis
