# Supplementary material for: Comprehensive analysis identifying aberrant DNA methylation in rectal mucosa from ulcerative colitis patients with neoplasia
Source: Oncotarget. 2018 Sep 4;9(69):33149–59. doi: 10.18632/oncotarget.26032 (PMC6145694; doi:10.18632/oncotarget.26032)
Supplement: Supplementary file 1 [file oncotarget-09-33149-s001.pdf]

Comprehensive analysis identifying aberrant DNA methylation in rectal mucosa from ulcerative colitis patients with neoplasia

SUPPLEMENTARY MATERIALS

Supplementary Table 1: Beta values of 11 DMRs in non-neoplastic rectal mucosa of UC and UC-CRC, and neoplastic tissues of UC-CRC

	Rectal (UC, *n* = 8)	Rectal (UC-CRC, *n* = 8)	Neoplasm (UC-CRC, *n* = 8)
SIX2	0.50 ± 0.11	0.63 ± 0.10[a]	0.68 ± 0.18
SATB2;SATB2-AS1	0.16 ± 0.07	0.21 ± 0.13	0.24 ± 0.21
HAND2	0.47 ± 0.04	0.52 ± 0.06	0.55 ± 0.12
GDNF	0.34 ± 0.07	0.39 ± 0.07	0.52 ± 0.17
PLCXD3	0.47 ± 0.10	0.55 ± 0.08	0.63 ± 0.11
HPSE2	0.20 ± 0.03	0.26 ± 0.07[a]	0.38 ± 0.15
TBX3	0.32 ± 0.06	0.40 ± 0.07[a]	0.48 ± 0.10
PAX9	0.32 ± 0.07	0.39 ± 0.08	0.47 ± 0.10
MEIS2	0.41 ± 0.11	0.46 ± 0.14	0.50 ± 0.13
SALL1	0.23 ± 0.05	0.30 ± 0.08	0.44 ± 0.15[c]
LGALS3BP	0.52 ± 0.09	0.38 ± 0.09[b]	0.34 ± 0.07

Values are expressed as mean ± s.d. Student’s *t*-test was applied.
[a]$p < 0.05$, UC rectal vs. UC-CRC rectal.
[b]$p < 0.01$, UC rectal vs. UC-CRC rectal.
[c]$p < 0.05$, UC-CRC rectal vs. UC-CRC neoplasm.

Supplementary Table 2: Patient characteristics in training cohort

Characteristic		Patients without cancer (*n* = 24)	Patients with cancer (*n* = 24)	*p*-value
Gender	Male	18	15	0.35
	Female	6	9	
Age at surgery for UC, y (range)		35 (17–73)	43 (18–77)	0.22
Disease type	Total	22	22	1
	Left side	2	2	

Supplementary Table 3: Patient characteristics in validation cohort

Characteristic		Patients without cancer (*n* = 8)	Patients with cancer (*n* = 8)	*p*-value
Gender	Male	4	5	0.62
	Female	4	3	
Onset age, y (range)		31.5 (19–73)	29 (16–63)	0.67
Age at surgery for UC, y (range)		41 (20–74)	48 (28–66)	0.56
Disease duration, y (range)		6.5 (0.75–16)	11 (0.4–21)	0.14
Disease severity	Mild	2	6	0.14
	Middle	6	2	
	Severe	0	0	
Disease type	Total	6	7	0.53
	Left side	2	1	
